# Trends and disparities in the burden of oral cancer in Southeast Asia, 1990 to 2023: a retrospective analysis based on the Global Burden of Disease 2023 data

**DOI:** 10.12701/jyms.2026.43.45

**Published:** 2026-07-08

**Authors:** Bowen Li, Fatihah Azhar, Rasheed Abdulsalam

**Affiliations:** School of Dentistry, Lincoln University College, Selangor, Malaysia

**Keywords:** Global burden of disease, Malaysia, Mouth neoplasms, Southeast Asia

## Abstract

**Background:**

Oral cancer remains a major global public health issue, particularly in Southeast Asia, where its incidence and mortality rates are the highest worldwide. Malaysia, although a part of Southeast Asia, possesses a more advanced healthcare system, offering a unique comparison for understanding regional disparities. This study aimed to evaluate and compare the burden of oral cancer in Malaysia and Southeast Asia from 1990 to 2023 and forecast future trends using the most recent Global Burden of Disease (GBD) 2023 data.

**Methods:**

Age-standardized incidence, mortality, and disability-adjusted life years (DALYs) were extracted from the GBD 2023 database. Joinpoint regression was applied to identify temporal shifts and estimate the annual percent change and average annual percent change. Autoregressive integrated moving average (ARIMA) models were used to predict future trends from 2024 to 2038.

**Results:**

Malaysia has exhibited a largely stable burden of oral cancer over the past three decades, with DALYs increasing by only 2.3% and incidence by 18.0%. In contrast, Southeast Asia experienced substantial increases in DALYs (20.9%), incidence (38.8%), and mortality (14.9%). Marked sex disparities persisted, particularly in Southeast Asia, where males had 1.6× to 2.3× higher burden than females. ARIMA forecasts indicate that Malaysia’s oral cancer burden will remain largely stable, whereas Southeast Asia is projected to experience significant increases through 2038.

**Conclusion:**

Malaysia’s relatively stable trends may reflect stronger public health interventions, including tobacco control and improved access to care. In contrast, the continuously rising burden in Southeast Asia underscores the persistent gaps in prevention, early detection, and treatment.

## Introduction

Oral cancer is the most common malignancy of the head and neck and is characterized by its aggressive nature and poor prognosis. Globally, more than 450,000 new cases of oral cancer are diagnosed each year; however, only 40% to 50% of patients survive beyond 5 years after diagnosis [1,2]. As a prevalent malignancy, oral cancer poses a significant public health challenge worldwide [3]. It not only impairs patient quality of life but also imposes a substantial socioeconomic burden [4]. Moreover, the incidence of oral cancer varies geographically, with a disproportionately higher burden observed in low- and middle-income countries than in high-income nations [5,6]. According to the World Health Organization (WHO) regional office, the Southeast Asia region has one of the highest incidences and mortality rates of oral cancer among the six WHO regions globally [7]. Comparing the burden and long-term trends of oral cancer in Southeast Asia and Malaysia is crucial for developing effective health policies and screening strategies. Malaysia serves as an informative comparator within Southeast Asia because, while sharing similar core risk factors for oral cancer (e.g., betel quid chewing and tobacco use) with the region, it possesses a stronger economy, more developed healthcare system, and stronger public health policies. This unique combination allows for the examination of whether superior socioeconomic and healthcare conditions might be associated with a lower oral cancer burden.

The Global Burden of Disease (GBD) 2023 report provides the most current and comprehensive data available, derived from national and regional cancer registries and vital registration systems, enabling an in-depth assessment of the oral cancer burden across different areas [8]. Monitoring the magnitude of this burden, along with its demographic, geographic, and temporal variations, is essential for guiding health planning, setting clinical priorities, and identifying key research gaps.

In the present study, we used GBD 2023 data to analyze and compare the age-standardized incidence, mortality, and disability-adjusted life years (DALYs) of oral cancer (International Classification of Diseases, 10th Revision [ICD-10] codes C00–C08) [9] in Malaysia and Southeast Asia from 1990 to 2023. We further applied joinpoint regression to identify temporal shifts and autoregressive integrated moving average (ARIMA) models to forecast trends until 2038 [10]. By comparing Malaysia, a more developed nation within the region, with the broader Southeast Asian average, we aimed to examine whether stronger socioeconomic and healthcare conditions might be associated with a lower oral cancer burden despite shared risk factors. These findings provide descriptive evidence that can inform policymaking and prevention strategies.

## Methods

**Ethics statement:** This study did not involve human or animal subjects and was based solely on publicly available literature; therefore, ethics approval was not required according to the guidelines of our institution.

### 1. Data source

We analyzed the overall trends in oral cancer in Malaysia and compared them with the regional averages for Southeast Asia. We used the most recent version of the GBD 2023 results released at the end of October 2025. Data were extracted for the GBD cause category “Lip and oral cavity cancer” (ICD-10 C00–C08). The selected measures were DALYs, incidence, and mortality, which were extracted as age-standardized rates (ASRs) per 100,000 population standardized to the GBD standard population. Estimates were extracted for all ages, for sex (male, female, and both combined), for Malaysia (country-level) and Southeast Asia (regional aggregate as defined by the GBD), and for the period 1990 to 2023. The point estimates and 95% uncertainty intervals (UIs) were extracted. Data were extracted in accordance with the GBD data extraction framework, selecting variables related to disease type, age, and burden metrics without applying additional inclusion or exclusion criteria. ASRs were used as primary indicators to enable comparisons over time and between populations, with rates standardized to the GBD standard population. All data were retrieved from the GBD 2023 results tool via the Institute for Health Metrics and Evaluation website (http://ghdx.healthdata.org/).

### 2. Statistical methods

Joinpoint regression analysis was conducted using version 5.2.0 of the Joinpoint Regression Program developed by the U.S. National Cancer Institute. A maximum of three joinpoints was permitted given the 34-year study period. Model selection was performed using the permutation test method with an overall significance level of α=0.05. The Joinpoint Regression Program estimates the annual percent change (APC) and average APC (AAPC). The APC represents the annual rate of change calculated for each segment between trend changes. This method was applied to analyze temporal trends in the incidence, mortality, and DALYs of oral cancer, and 95% confidence intervals (CIs) were calculated. The AAPC provides an overall summary of the trends throughout the study period by averaging the APCs across all segments. In this analysis, the year was used as the independent variable, and the age-standardized incidence, mortality, and DALYs were treated as dependent variables. The model assumed a constant rate of change within each segment. If the AAPC is positive and its 95% CI does not include zero, the trend is classified as increasing; conversely, if the AAPC is negative and its CI does not include zero, the trend is classified as decreasing.

Statistical significance for the APC in each segment and for the overall AAPC was assessed using a significance level of α=0.05 (two-sided). Joinpoints were selected using the permutation test method with an overall significance level of 0.05, as implemented in the Joinpoint Regression Program. ARIMA models were implemented using R software (version 4.2.0; R Foundation for Statistical Computing, Vienna, Austria) to forecast future trends in age-standardized incidence, mortality, and DALY rates for oral cancer from 2024 to 2038. Before model fitting, the stationarity of each time series was examined using the augmented Dickey-Fuller test. For non-stationary series, differencing (d=1) was applied to achieve stationarity. Model identification and selection were performed using the automatic ARIMA algorithm based on the Akaike Information Criterion (AIC). The parameters selected for the final models were as follows: for Malaysia, ARIMA (0,1,1) for DALYs, ARIMA (1,1,0) for incidence, and ARIMA (0,1,1) for mortality; for Southeast Asia, ARIMA (0,1,1) for DALYs, ARIMA (1,1,1) for incidence, and ARIMA (0,1,1) for mortality. All models were checked for residual autocorrelation using the Ljung-Box test to ensure an adequate fit. The 95% CIs for the forecasts were generated based on the standard errors of the model estimates. When the optimal model selected by the AIC was a random walk (i.e., ARIMA (0,1,0)) or a model with d=1 and no significant autoregressive or moving average terms, multi-step ahead forecasts reverted to the last observed value, producing constant projections. This reflected the inherent unpredictability of the series, based on historical dynamics. The ARIMA models were fitted using point estimates of ASRs. The 95% CI reported in the forecast tables were generated from the standard errors of the model estimates and not from the GBD UIs.

## Results

### 1. Time trends in disability-adjusted life years

Overall, the age-standardized DALY rate in Malaysia remained relatively stable, increasing only slightly from 39.5 in 1990 to 40.4 in 2023, representing a growth of only 2.3% over the past three decades. In contrast, Southeast Asia showed a clear upward trend, with the age-standardized DALY rate increasing from 53.27 in 1990 to 64.38 in 2023, an overall increase of 20.9% over the past three decades, indicating a substantial increase in disease burden (Table 1).

Marked sex disparities persisted in both regions. In Malaysia, male DALY rates were approximately 1.3× to 1.4× higher than female DALY rates (42.2–47.2 vs. 30.8–38.5). In Southeast Asia, the disparity was larger, with male DALY rates 2.2× to 2.3× higher than female DALY rates (72.2–90.7 vs. 36.0–40.1) (Table 1). Overall and sex-specific DALY rates in Southeast Asia consistently exceeded those in Malaysia. Regarding recent changes, Malaysia experienced a sharp increase in 2022, followed by a decline in 2023, while Southeast Asia continued its upward trajectory (Fig. 1).

### 2. Time trends in incidence

The incidence rate in Malaysia showed a modest increase over the past three decades, from 2.61 in 1990 to 3.08 in 2023 (18.0% increase), whereas Southeast Asia experienced a much larger rise, from 3.22 to 4.47 (38.8% increase) (Table 2). Notably, sex differences in Malaysia were minimal, with female incidence even slightly exceeding that in males (2.70–3.14 vs. 2.42–3.11), a pattern contrasting with the pronounced male predominance observed in Southeast Asia, where male incidence was approximately 1.6× to 1.7× higher than that in females (3.92–5.67 vs. 2.58–3.37) (Table 2). As shown in Fig. 2, the rising trend in Southeast Asia was predominantly driven by males, whereas Malaysia exhibited relatively stable trends with minimal sex divergence. In recent years, Malaysia’s incidence peaked at 3.13 in 2022 and declined in 2023, while that in Southeast Asia continued to steadily increase.

### 3. Time trends in mortality

In Malaysia, the mortality rate showed a slow, long-term decline from 1.73 in 1990 to 1.70 in 2023 (a 1.7% reduction), despite an anomalous peak of 1.75 in 2022. In contrast, Southeast Asia exhibited a continuous upward trend, with the mortality rate increasing from 2.08 to 2.39 (a 14.9% increase) over the same period (Table 3). The overall mortality rate in Southeast Asia was approximately 40% higher than that in Malaysia, representing the largest regional disparity among the three burden indicators. Sex differences in Malaysia were minimal, whereas males experienced approximately 2.0× to 2.1× higher mortality than females in Southeast Asia (2.73–3.28 vs. 1.51–1.64) (Table 3). As shown in Fig. 3, Malaysia’s mortality rate declined steadily until 2020, rebounded sharply in 2022, and declined again in 2023, whereas Southeast Asia’s mortality rate continued to rise over the past 3 years.

### 4. Joinpoint regression analysis

#### 1) Trends in Malaysia

Joinpoint regression analysis showed that the DALYs, incidence, and mortality of oral cancer in Malaysia exhibited only modest overall changes between 1990 and 2023 (Fig. 4). DALYs showed a modest increase overall (AAPC, 0.15), with three distinct phases: an increase from 1990 to 2001 (APC, 0.45), a decline from 2002 to 2019 (APC, –0.54), and a rebound from 2020 to 2023 (APC, 3.15). The female rates remained stable (AAPC, 0.04), whereas the male rates increased, particularly between 2021 and 2023 (APC, 4.5) (Fig. 4A). Incidence showed a mild increase overall (AAPC, 0.57), with an increase from 1990 to 2003 (APC, 0.81), a decline from 2004 to 2019 (APC, –0.36), and a subsequent increase after 2020 (APC, 4.53). The increase was smaller in females (AAPC, 0.48) than in males (AAPC, 0.73) (Fig. 4B).

Overall, the mortality rate remained stable (AAPC, 0.08). It increased slightly from 1990 to 2001 (APC, 0.64), decreased from 2002 to 2019 (APC, –0.97), and increased again after 2020 (APC, 4.23). The female rates declined slightly (AAPC, –0.08), whereas the male rates showed a modest increase (AAPC, 0.25) (Fig. 4C).

#### 2) Trends in Southeast Asia

In Southeast Asia, DALYs showed a clear overall increase (AAPC, 0.63), with several shifts from 1990 to 2023 (Fig. 4D). Rapid increases were observed in 1990 to 1994 (APC, 1.64), 1997 to 1999 (APC, 1.90), and 2019 to 2023 (APC, 1.80), whereas only brief declines occurred between 1994 and 1996. Females showed a mild increase (AAPC, 0.35), whereas males showed a much faster increase (AAPC, 0.74).

Incidence increased continuously (AAPC, 1.04), with rises occurring from 1990 to 1993 (APC, 2.30), 1997 to 1999 (APC, 1.79), and 2019 to 2023 (APC, 2.81). The female rates increased gradually (AAPC, 0.83), whereas the male rates increased more rapidly (AAPC, 1.16) (Fig. 4E).

The overall mortality rate has also increased (AAPC, 0.46). After initial fluctuations— an increase in 1990 to 1993, a decline in 1994 to 1996, and another increase in 1997 to 2000—the rates remained stable from 2001 to 2018, followed by an increase after 2019 (APC, 1.55). The female rates increased modestly (AAPC, 0.20), with a decline from 2000 to 2018, whereas the male rates increased more steeply (AAPC, 0.61) (Fig. 4F).

### 5. Oral cancer autoregressive integrated moving average forecasts

In Malaysia, the age-standardized DALY rate showed relative stability historically, and forecasts suggest that it will remain stable from 2023 to 2038 at approximately 40.41. The nearly constant projections indicate that the optimal ARIMA model identified for each indicator selected a unit root (d=1) with negligible trend components, meaning that the best estimate for future years is the last observed value given the lack of a strong historical trend. In Malaysia, the incidence has shown a relatively stable increase in the past, and forecasts suggest that the incidence rate will remain stable or increase only slightly. The mortality rate has shown a relatively stable decrease in the past, and the forecast suggests that from 2023 to 2038, the value will remain near the current level at approximately 1.70 (Table 4, Fig. 5).

In Southeast Asia, the age-standardized DALY rate attributable to oral cancer has increased markedly over the past three decades and is projected to continue rising from 64.38 in 2023 to 69.43 in 2038. The projected incidence showed a pattern similar to that of the DALY rate, increasing from 4.47 in 2023 to 5.03 in 2038, suggesting that the oral cancer incidence rate will continue to rise significantly over the next 15 years. The mortality rate in Southeast Asia is also projected to exhibit a continuous and notable upward trend, increasing from 2.39 in 2023 to an estimated 2.53 in 2038 (Table 5, Fig. 6).

## Discussion

The global oral cancer burden has increased since 1990. With rising healthcare expenditure and socioeconomic costs, oral cancer has become a major public health concern worldwide. Given its poor prognosis and substantial impact on patient quality of life, greater attention and further research are urgently needed [11]. Comparing Malaysia with the broader Southeast Asian region helps clarify Malaysia’s relative disease burden and highlights differences in long-term trends, providing an important context for interpreting national patterns. This comparison also helps to identify potential gaps in prevention and control efforts, offering descriptive evidence that may help inform more targeted public health strategies. Malaysia ranks in the upper-middle tier among the 11 Southeast Asian countries, trailing only Singapore and Brunei, making it one of the region’s more developed nations [12].

The persistently high incidence of oral cancer in Southeast Asia is closely related to the socioeconomic context. In some countries, rural and low-income populations have limited health literacy and insufficient awareness of the carcinogenic dangers of betel quid and tobacco. Deep-rooted traditional practices and social customs make betel quid chewing prevalent and difficult to eradicate in these groups [13]. Comparison of Malaysia with the broader Southeast Asian region provides a useful regional comparator. As part of Southeast Asia, Malaysia shares similar environmental and core risk factors for oral cancer with neighboring countries, ensuring comparability in etiology. Through this comparison, it is possible to examine whether, against a shared background of risk factors, superior socioeconomic conditions, and more proactive public health interventions may help reduce the oral cancer epidemic.

### 1. Disability-adjusted life years

This study revealed that Malaysia’s DALY rates remained relatively stable, whereas Southeast Asia demonstrated a marked upward trend, with an overall increase of 20.9% in DALYs over the past three decades, indicating a substantial increase in disease burden. Malaysia’s stable trend is unlikely to reflect the disappearance of oral cancer risk factors but may be associated with sustained public health policies, including progressively strengthened tobacco and alcohol control measures such as tax increases, smoking bans in public places, and advertising restrictions [14]. The continued increase in DALY rates in Southeast Asia, notably 2× to 3× higher than that in Malaysia, may be linked to disparities in policy enforcement, with many countries facing insufficient funding and limited governance capacity. In some nations, economic interests tied to the betel quid industry may further hinder strong regulatory actions [15].

In both Malaysia and the broader Southeast Asian region, DALYs for males were significantly higher than those for females, likely because of the higher frequency and intensity of exposure to major risk factors for oral cancer in males. In Malaysia and Southeast Asia, the practice of chewing betel quid showed marked differences by sex. This behavior is more prevalent among men and is often regarded as a traditional social activity, a method of staying alert, or even a reflection of masculinity [16]. Historically, the smoking rate has been considerably higher among men than among women [17]. Tobacco is a strong independent risk factor for oral cancer and has a synergistic carcinogenic effect when combined with betel quid.

### 2. Incidence

In the sex-stratified analysis, the sex difference in Malaysia was minimal, whereas in Southeast Asia, the incidence rate among males was approximately 1.7× that among females. The comparatively higher incidence among Malaysian females is noteworthy and contradicts traditional expectations. One possible explanation is that the participation rate of the female labor force is relatively high in Malaysia, leading to greater exposure to shared etiological risk factors for oral cancer [18]. Another explanation is that community-based health-screening programs targeting women may be more widespread in Malaysia, resulting in incidental detection of oral cancer or precancerous lesions during screening, thereby increasing the reported incidence among females [19].

In contrast, the markedly higher incidence of oral cancer among males in Southeast Asia may be primarily associated with a high prevalence of smoking, betel quid chewing, and alcohol consumption. These carcinogenic exposures directly affect the oral mucosa and represent primary risk factors for oral cancer. Moreover, men were more likely to engage in the combined high-risk behaviors of smoking, alcohol consumption, and betel quid chewing. The carcinogenic effects of these behaviors are not merely additive but synergistic [20,21].

### 3. Mortality rate

In Malaysia, the mortality rate showed a long-term gradual decline before 2021, although an unusual increase was observed in 2022, which may have been influenced by external factors. In contrast, Southeast Asia exhibited a consistently rising trend, with an overall increase of 14.9% over the past three decades.

The sustained decline in Malaysia’s mortality rate may be associated with the strengthening of its healthcare safety net. With improvements in the nation’s overall healthcare capacity, potentially malignant oral disorders and cancers are more likely to be detected at earlier stages. In addition, the population has better access to standardized treatments, including surgery, radiotherapy, and chemotherapy, which directly improve patient survival outcomes [22].

In contrast, Southeast Asia faces significant shortages in healthcare resources, particularly oncology services, which are often concentrated in a few major cities. Rural and low-income populations, especially in the remote areas of Laos, Myanmar, and Cambodia, have limited access to timely diagnosis and treatment. Consequently, many patients are diagnosed at a later stage, leading to poor treatment outcomes and higher mortality rates. Additionally, cancer care is often inconsistent or incomplete, further contributing to higher disability and mortality burdens [23]. This further indicates that in certain parts of Southeast Asia, the availability and accessibility of oncology care have remained limited in recent years.

### 4. Joinpoint regression analysis

According to the Jointpoint regression analysis, the temporal trend of oral cancer DALYs in Malaysia from 1990 to 2023 showed significant phase-specific changes. Overall, the DALYs increased from 1990 to 2001, followed by a prolonged decline from 2002 to 2019, but rebounded rapidly after 2020. The sharp rebound after 2020 coincided with the coronavirus disease 2019 (COVID-19) pandemic [24]. The increase in incidence during the pandemic was greater than the increase in DALY rates, indicating that the surge in new cases was the primary driver of the escalating disease burden. The sharp increase in mortality after 2020 suggests that the pandemic had a stronger impact on mortality rates, reflecting the severe consequences of accumulated late-stage cases. The findings from 2002 to 2019, prior to the COVID-19 outbreak in Malaysia, were consistent with a reduction in mortality and notable treatment improvements among females [25].

### 5. Autoregressive integrated moving average forecast

The ARIMA forecast was generated under the assumption that future trends would follow past patterns and that no major changes would occur [26]. Therefore, the predictions made in this study reflect the trends that may emerge if no additional interventions or unexpected events occur. Although Malaysia’s DALY rates, incidence, and mortality rates increased after 2019, coinciding with the COVID-19 pandemic, this pattern likely reflects temporary disruptions in cancer care and subsequent catch-up diagnoses, rather than a sustained change in the underlying disease burden [27].

In contrast, the age-standardized incidence rate, DALYs, and mortality rate in Southeast Asia showed a persistent and substantial upward trend from the historical period to the projected future, indicating a continuously increasing burden of oral cancer in this region. This ongoing increase suggests that the current primary prevention measures are inadequate or ineffective. The continuously growing incidence implies that more patients will require diagnosis and treatment in the future, placing mounting pressure on healthcare systems. Malaysia and Southeast Asia share broadly similar etiological risk profiles for oral cancer; however, the observed outcomes differ markedly between the two regions. These findings indicate that public health interventions implemented at the national level could play a substantial role in oral cancer prevention and control. Malaysia’s strong tobacco control policies implemented after 2000 corresponded to a more pronounced decline in incidence compared with that of the overall Southeast Asian region. This temporal correspondence is consistent with the possibility that these policies contributed to the observed differences, although causal inferences cannot be drawn from ecological data. These findings can help international organizations, such as the WHO, as well as national governments to allocate resources more effectively and implement targeted prevention strategies [28,29].

The major drivers of oral cancer in Southeast Asia are smoking, alcohol consumption, and betel quid chewing. This is particularly evident in Indonesia, Myanmar, Laos, and regions of Thailand and the Philippines, where the prevalence of male smoking is extremely high, exceeding 60% in some countries [30,31]. The coexistence of multiple high-risk behaviors further increases the risk of developing oral cancer. This highlights the importance of strengthening tobacco and betel quid control policies. Within the limitations of this ecological analysis, to effectively control the future burden of oral cancer, Southeast Asia may need to consider stricter measures.

This study had several limitations. First, because it is based on aggregated ecological-level data from the GBD study, direct causal relationships between specific policies or interventions and the observed disease burden trends cannot be established. The associations discussed throughout the Discussion should therefore be interpreted as hypothesis-generating patterns that are consistent with the existing policy context, rather than as causal attributions. Second, Southeast Asia is highly heterogeneous in terms of socioeconomic status and risk factor profiles (e.g., tobacco use, betel quid chewing). Our analysis may have obscured important cross-country differences by aggregating data across regions. Therefore, the regional averages presented here should be interpreted as general patterns rather than being directly applicable to every country in Southeast Asia. Although the most recent GBD database was used, the COVID-19 pandemic from 2020 to 2022 may have disrupted data collection and survey methods, potentially affecting the accuracy of estimates during this period. In addition, issues related to the quality and collection of primary data remain, particularly in remote areas where accurate oral cancer information is difficult to obtain. Methodological deficiencies and possible underreporting are recurrent limitations of the GBD framework. Moreover, owing to current data constraints, it was not possible to conduct separate analyses of lip, buccal, and oral cavity cancers. Finally, our ARIMA forecasts were fitted using only GBD point estimates and the reported 95% CIs were derived from model standard errors rather than from GBD UIs. This approach does not account for measurement errors inherent in the GBD data, potentially underestimating the forecast uncertainty. Future studies could address this issue by incorporating the upper and lower bounds of the GBD UIs into the forecasting framework.

In summary, the comparison between Malaysia and Southeast Asia highlights divergent oral cancer burden trends despite shared etiological factors, suggesting that national-level public health interventions may play a role in prevention and control. Southeast Asian countries should consider strengthening tobacco and betel quid control policies, expanding early screening programs, enhancing oncology care capacity, and establishing comprehensive surveillance systems to curb the increasing burden of oral cancer. These recommendations are made considering the ecological nature of the evidence presented.

## Figures and Tables

**Fig. 1.**
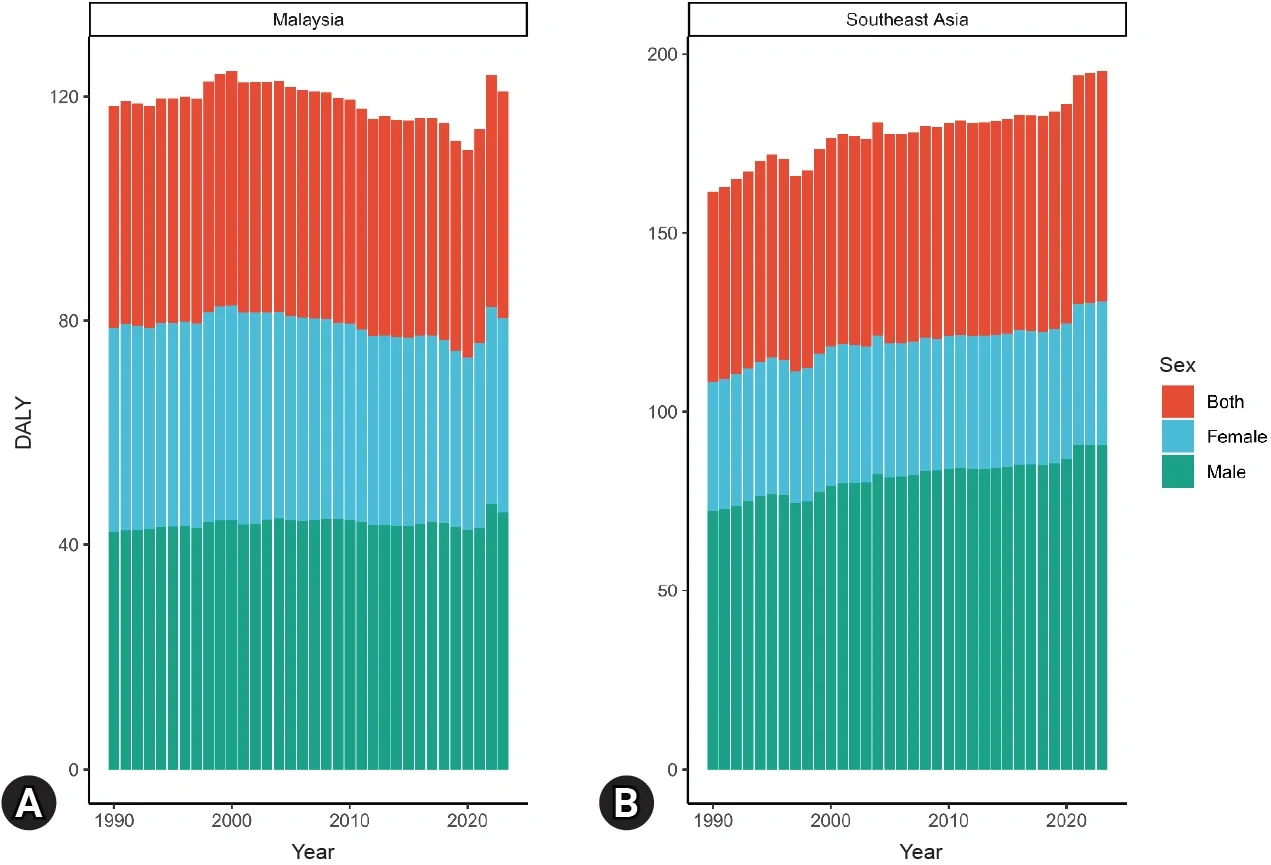
Trends in age-standardized disability-adjusted life years for oral cancer in Malaysia and Southeast Asia, 1990 to 2023. The figure presents the age-standardized disability-adjusted life years (DALYs) per 100,000 population for oral cancer in (A) Malaysia and (B) Southeast Asia from 1990 to 2023. Data are shown for the total population (“Both”), males, and females. “Both” represents the age-standardized DALY rate for the total population (males and females combined). It is not the sum of male and female rates, but rather the overall population estimate derived from the Global Burden of Disease 2023 database.

**Fig. 2.**
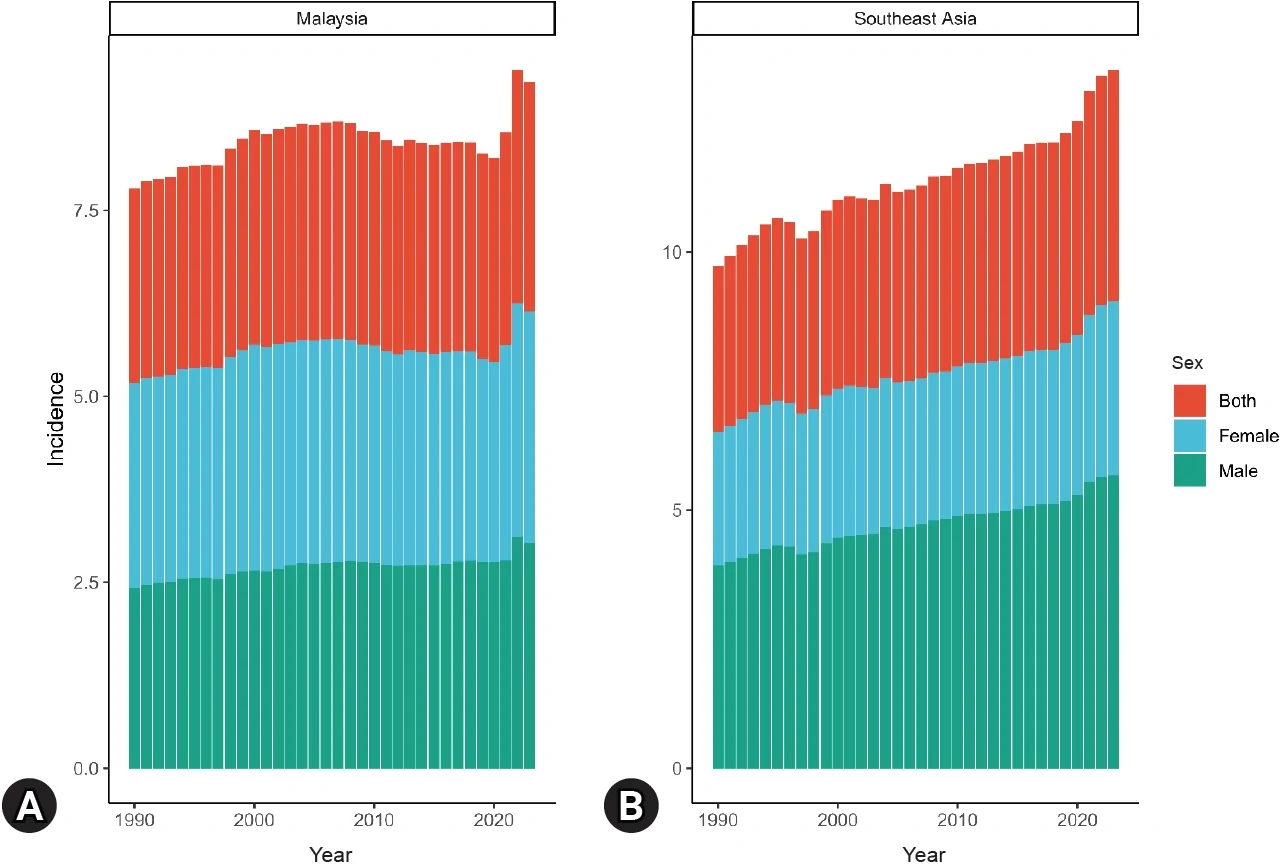
Trends in age-standardized incidence rates of oral cancer in Malaysia and Southeast Asia, 1990 to 2023. The figure presents the age-standardized incidence rates (per 100,000 population) of oral cancer in (A) Malaysia and (B) Southeast Asia from 1990 to 2023. Data are shown for the total population (“Both”), males, and females. “Both” represents the age-standardized incidence rate for the total population (males and females combined). It is the overall population estimate derived from the Global Burden of Disease 2023 database and is not the sum of the male and female rates.

**Fig. 3.**
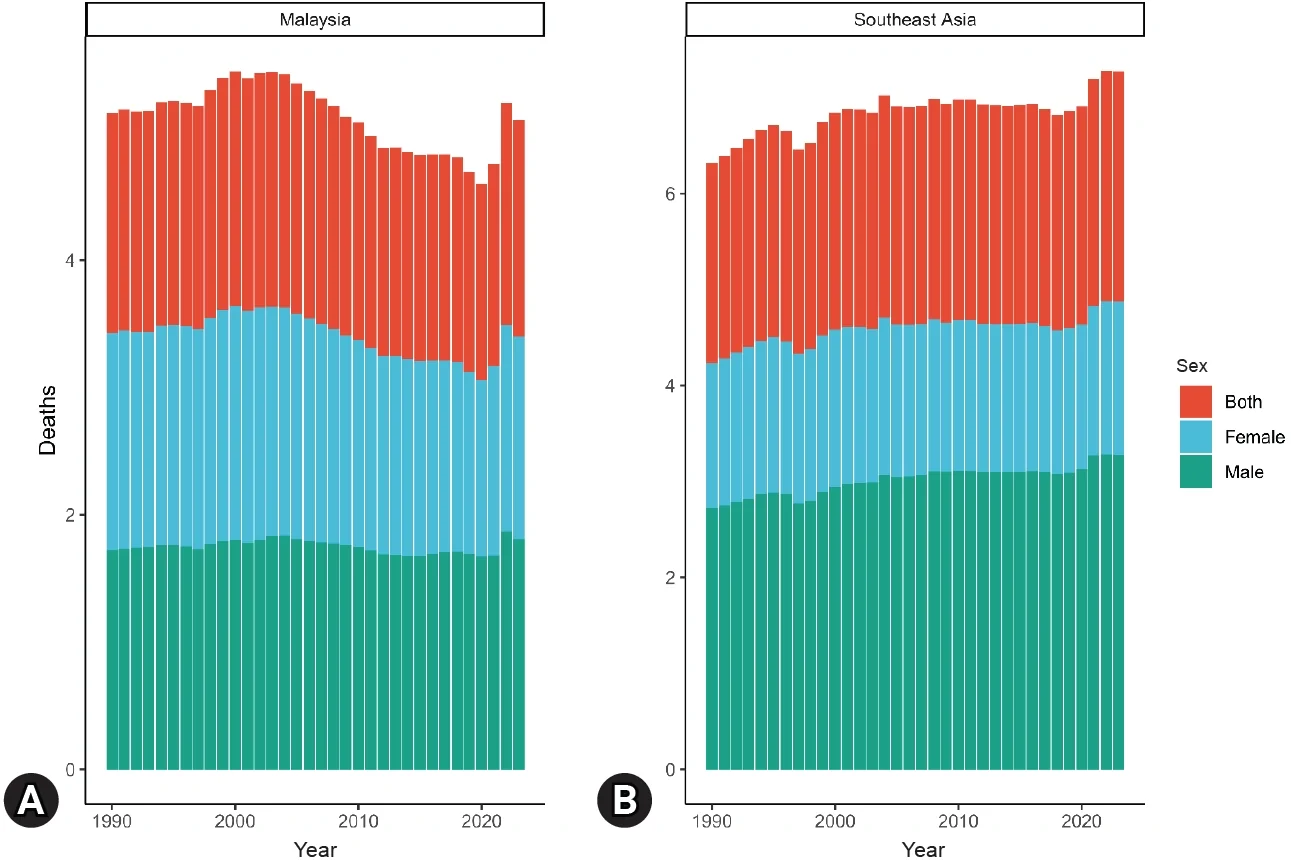
Trends in age-standardized mortality rates of oral cancer in Malaysia and Southeast Asia, 1990 to 2023. The figure presents the age-standardized mortality rates (per 100,000 population) of oral cancer in (A) Malaysia and (B) Southeast Asia from 1990 to 2023. Data are shown for the total population (“Both”), males, and females. “Both” represents the age-standardized mortality rate for the total population (males and females combined). It is the overall population estimate derived from the Global Burden of Disease 2023 database and is not the sum of the male and female rates.

**Fig. 4.**
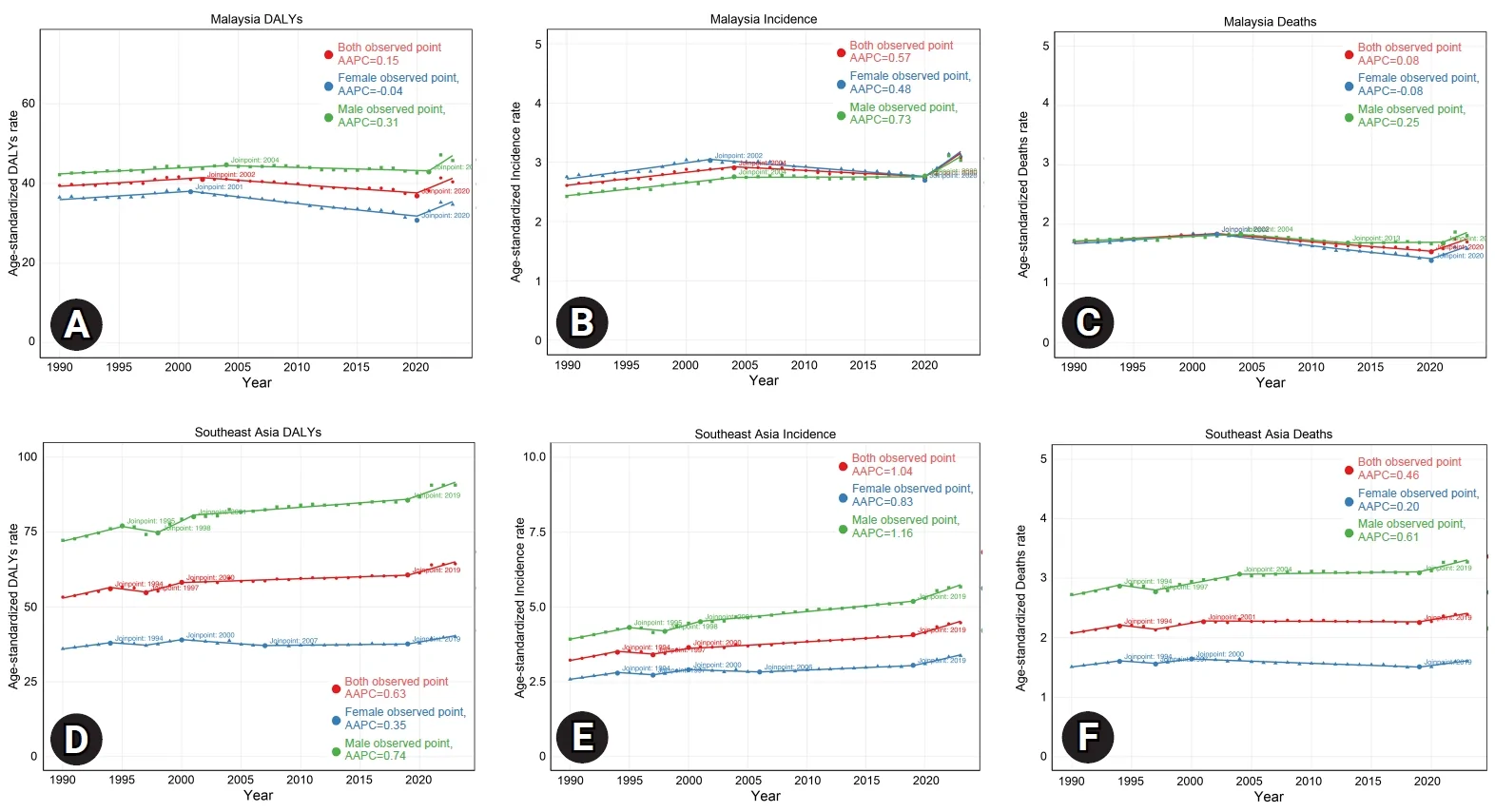
Oral cancer trends in age-standardized disability-adjusted life year rates, incidence rates, and mortality rates in Malaysia and Southeast Asia, 1990 to 2023, analyzed by joinpoint regression. (A) Malaysia disability-adjusted life year (DALY) rates, (B) Malaysia incidence rates, (C) Malaysia mortality rates, (D) Southeast Asia DALY rates, (E) Southeast Asia incidence rates, and (F) Southeast Asia mortality rates. The annual percentage change (APC) and average APC (AAPC) are provided for distinct periods. *p<0.05, significant change.

**Fig. 5.**
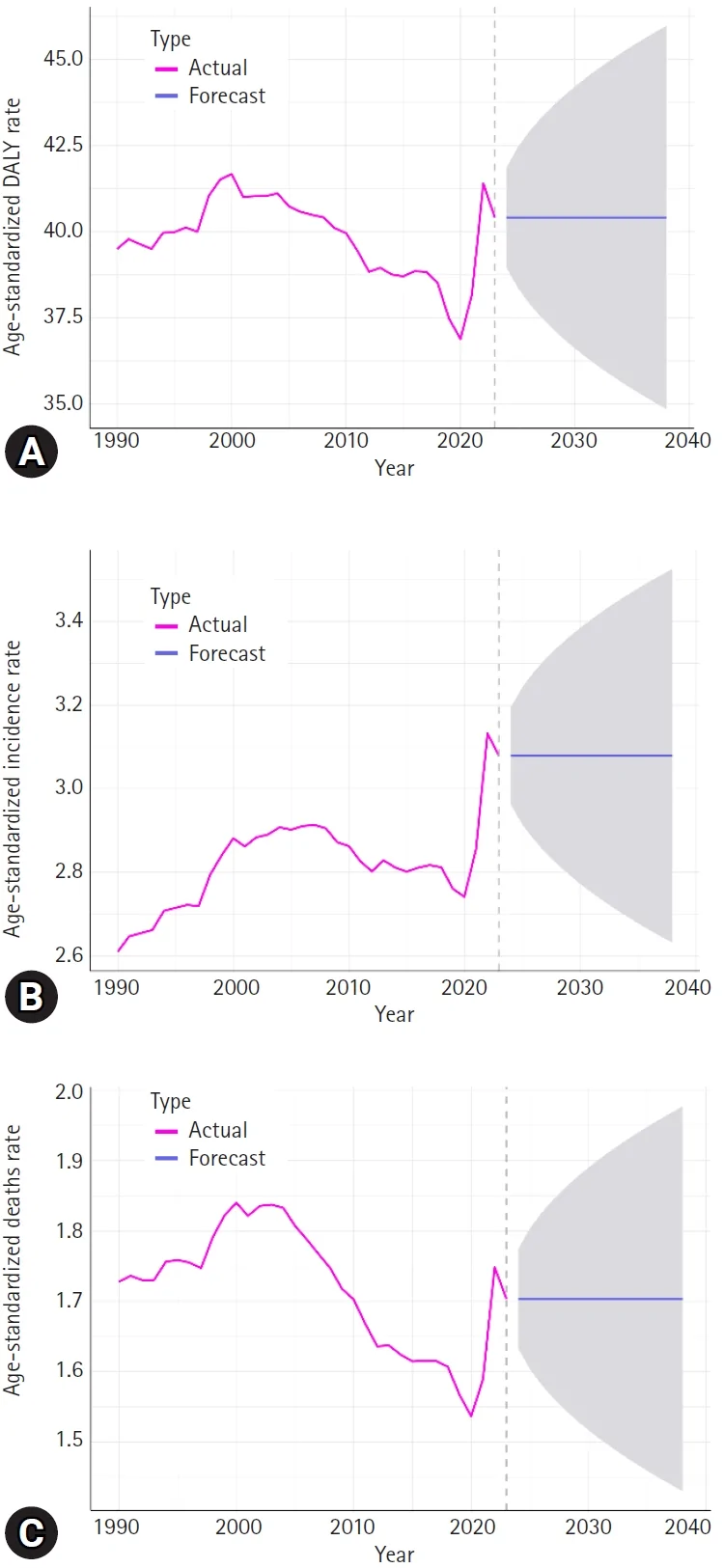
Graph of oral cancer trends in Malaysia from 1990 to 2038 using the autoregressive integrated moving average model. (A) Disability-adjusted life year (DALY) rates, (B) incidence rates, and (C) mortality rates. The red line indicates observed values, the blue line indicates forecasts, and the shaded area represents the 95% confidence interval. The period from 1990 to 2023 represents the observed historical trends, while the period from 2024 to 2038 represents the projected future trends.

**Fig. 6.**
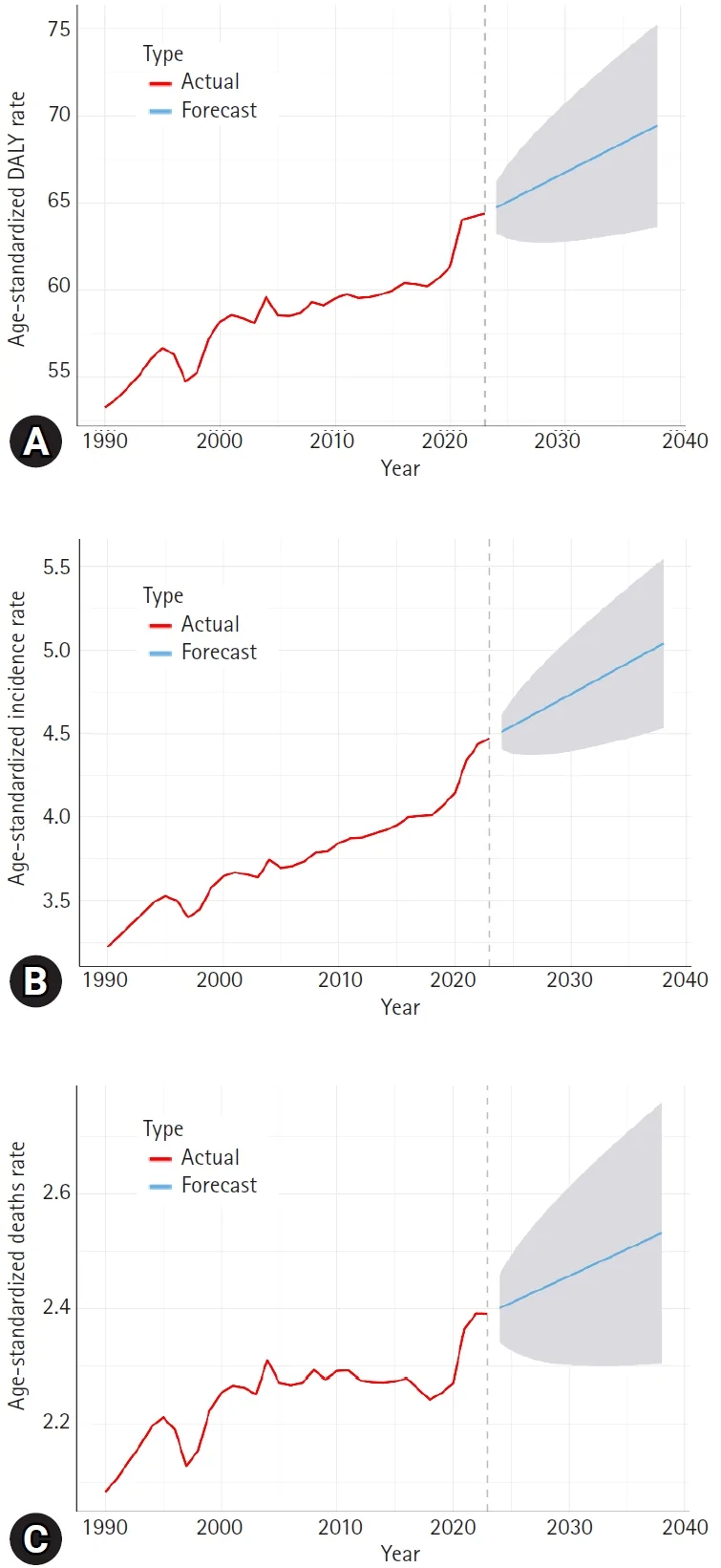
Graph of oral cancer trends in Southeast Asia from 1990 to 2038 using the autoregressive integrated moving average model. (A) Disability-adjusted life year (DALY) rates, (B) incidence rates, and (C) mortality rates. The red line indicates observed values, the blue line indicates forecasts, and the shaded area represents the 95% confidence interval. The period from 1990 to 2023 represents the observed historical trends, while the period from 2024 to 2038 represents the projected future trends.

**Table 1.** Age-standardized DALY rates for lip and oral cavity cancer (per 100,000 population): Malaysia vs. Southeast Asia, 1990–2023

Region	Year	Overall		Male		Female	
		Mean (95% UI)	Growth (%)	Mean (95% UI)	Growth (%)	Mean (95% UI)	Growth (%)
Malaysia	1990	39.50 (29.67–52.24)	-	42.23 (28.49–60.30)	-	36.55 (25.30–51.96)	-
	2020	36.89 (30.55–43.67)	–6.6	42.66 (33.84–52.28)	+1.0	30.78 (24.16–41.15)	–15.8
	2021	38.18 (31.68–45.66)	–3.3	42.91 (33.18–53.20)	+1.6	33.10 (25.31–44.00)	–9.4
	2022	41.40 (34.44–49.45)	+4.8	47.18 (34.64–58.87)	+11.7	35.26 (25.84–49.48)	–3.5
	2023	40.41 (32.70–49.81)	+2.3	45.77 (33.33–58.84)	+8.4	34.73 (24.61–47.77)	–5.0
Southeast Asia	1990	53.27 (42.82–67.33)	-	72.22 (52.08–98.47)	-	36.01 (26.45–48.93)	-
	2020	61.33 (50.47–75.36)	+15.1	86.70 (68.27–110.14)	+20.1	37.90 (30.31–50.16)	+5.2
	2021	64.02 (52.21–78.27)	+20.2	90.64 (70.10–117.61)	+25.5	39.47 (31.68–52.77)	+9.6
	2022	64.22 (53.16–77.35)	+20.6	90.65 (70.10–115.77)	+25.5	39.80 (31.38–53.39)	+10.5
	2023	64.38 (53.45–77.61)	+20.9	90.69 (69.79–113.42)	+25.6	40.05 (31.28–54.79)	+11.2

Growth (%) is calculated relative to the 1990 baseline value. DALY, disability-adjusted life year; UI, uncertainty interval.

**Table 2.** Age-standardized incidence rates for lip and oral cavity cancer (per 100,000 population): Malaysia vs. Southeast Asia, 1990-2023

Region	Year	Overall		Male		Female	
		Mean (95% UI)	Growth (%)	Mean (95% UI)	Growth (%)	Mean (95% UI)	Growth (%)
Malaysia	1990	2.61 (1.94–3.47)	-	2.42 (1.62–3.55)	-	2.76 (1.93–4.00)	-
	2020	2.74 (2.24–3.38)	+5.0	2.77 (2.15–3.52)	+14.5	2.70 (2.08–3.66)	-2.2
	2021	2.85 (2.34–3.53)	+9.4	2.79 (2.14–3.54)	+15.3	2.90 (2.16–3.97)	+5.1
	2022	3.13 (2.52–3.90)	+20.0	3.11 (2.27–4.01)	+28.5	3.14 (2.23–4.47)	+13.8
	2023	3.08 (2.46–3.92)	+18.0	3.03 (2.18–4.01)	+25.2	3.11 (2.19–4.43)	+12.7
Southeast Asia	1990	3.22 (2.53–4.05)	-	3.92 (2.79–5.33)	-	2.58 (1.91–3.55)	-
	2020	4.14 (3.40–5.01)	+28.6	5.29 (4.18–6.82)	+35.0	3.10 (2.42–4.06)	+20.2
	2021	4.34 (3.51–5.39)	+34.8	5.54 (4.32–7.15)	+41.3	3.24 (2.51–4.35)	+25.6
	2022	4.44 (3.61–5.43)	+37.9	5.64 (4.37–7.35)	+43.9	3.33 (2.55–4.49)	+29.1
	2023	4.47 (3.68–5.51)	+38.8	5.67 (4.39–7.35)	+44.6	3.37 (2.54–4.64)	+30.6

Growth (%) is calculated relative to the 1990 baseline value. UI, uncertainty interval.

**Table 3.** Age-standardized death rates for lip and oral cavity cancer (per 100,000 population): Malaysia vs. Southeast Asia, 1990–2023

Region	Year	Overall		Male		Female	
		Mean (95% UI)	Growth (%)	Mean (95% UI)	Growth (%)	Mean (95% UI)	Growth (%)
Malaysia	1990	1.73 (1.29–2.30)	-	1.72 (1.16–2.44)	-	1.70 (1.16–2.49)	-
	2020	1.54 (1.27–1.84)	–11.0	1.67 (1.32–2.06)	–2.9	1.39 (1.10–1.88)	–18.2
	2021	1.59 (1.32–1.92)	–8.1	1.68 (1.29–2.09)	–2.3	1.48 (1.12–2.01)	–12.9
	2022	1.75 (1.45–2.09)	+1.2	1.87 (1.38–2.34)	+8.7	1.62 (1.16–2.27)	–4.7
	2023	1.70 (1.39–2.10)	–1.7	1.81 (1.32–2.32)	+5.2	1.59 (1.11–2.23)	–6.5
Southeast Asia	1990	2.08 (1.65–2.61)	-	2.73 (1.95–3.72)	-	1.51 (1.12–2.04)	-
	2020	2.27 (1.90–2.75)	+9.1	3.13 (2.48–3.94)	+14.7	1.51 (1.23–1.98)	+0.0
	2021	2.37 (1.95–2.87)	+13.7	3.27 (2.55–4.21)	+19.8	1.56 (1.26–2.05)	+3.3
	2022	2.39 (2.00–2.88)	+14.9	3.28 (2.56–4.16)	+20.1	1.60 (1.26–2.12)	+6.0
	2023	2.39 (2.01–2.87)	+14.9	3.27 (2.53–4.09)	+19.8	1.60 (1.27–2.16)	+6.0

Growth (%) is calculated relative to the 1990 baseline value. UI, uncertainty interval.

**Table 4.** Forecasted age-standardized DALYs, incidence, and death rates for oral cancer (per 100,000 population) from 2023 to 2038 in Malaysia

Year	DALYs (95% CI)	Incidence (95% CI)	Death (95% CI)
2023	40.41 (32.70–49.81)	3.07 (2.46–3.92)	1.70 (1.39–2.10)
2024	40.41 (37.20–43.62)	3.07 (2.82–3.33)	1.70 (1.54–1.86)
2025	40.41 (36.89–43.93)	3.07 (2.79–3.36)	1.70 (1.53–1.87)
2026	40.41 (36.61–44.21)	3.07 (2.77–3.38)	1.70 (1.51–1.89)
2027	40.41 (36.35–44.47)	3.07 (2.75–3.40)	1.70 (1.50–1.90)
2028	40.41 (36.10–44.72)	3.07 (2.73–3.42)	1.70 (1.49–1.91)
2029	40.41 (35.87–44.95)	3.07 (2.71–3.44)	1.70 (1.48–1.92)
2030	40.41 (35.65–45.17)	3.07 (2.69–3.46)	1.70 (1.46–1.93)
2031	40.41 (35.44–45.38)	3.07 (2.67–3.47)	1.70 (1.45–1.94)
2032	40.41 (35.23–45.58)	3.07 (2.66–3.49)	1.70 (1.44–1.95)
2033	40.41 (35.04–45.78)	3.07 (2.64–3.51)	1.70 (1.43–1.96)
2034	40.41 (34.85–45.97)	3.07 (2.63–3.52)	1.70 (1.42–1.97)
2035	40.41 (38.97–41.84)	3.07 (2.96–3.19)	1.70 (1.62–1.77)
2036	40.41 (38.38–42.44)	3.07 (2.91–3.24)	1.70 (1.60–1.80)
2037	40.41 (37.92–42.90)	3.07 (2.78–3.27)	1.70 (1.58–1.82)
2038	40.41 (37.54–43.28)	3.07 (2.84–3.30)	1.70 (1.56–1.84)

DALY, disability-adjusted life year; CI, confidence interval.

**Table 5.** Forecasted age-standardized DALYs, incidence, and death rates for oral cancer (per 100,000 population) from 2023 to 2038 in Southeast Asia

Year	DALYs (95% CI)	Incidence (95% CI)	Death (95% CI)
2023	64.38 (53.45–77.61)	4.47 (3.68–5.51)	2.39 (2.01–2.87)
2024	64.72 (62.70–69.42)	4.50 (4.37–4.94)	2.40 (2.30–2.56)
2025	65.05 (62.72–70.08)	4.54 (4.38–5.01)	2.41 (2.30–2.59)
2026	65.39 (62.76–70.71)	4.58 (4.39–5.07)	2.41 (2.30–2.61)
2027	65.73 (62.82–71.32)	4.62 (4.40–5.13)	2.42 (2.30–2.63)
2028	66.06 (62.90–71.92)	4.65 (4.42–5.20)	2.43 (2.30–2.65)
2029	66.40 (63.00–72.50)	4.69 (4.43–5.26)	2.44 (2.29–2.67)
2030	66.74 (63.10–73.07)	4.73 (4.45–5.31)	2.45 (2.30–2.68)
2031	67.07 (63.22–73.63)	4.77 (4.47–5.37)	2.46 (2.30–2.70)
2032	67.41 (63.34–74.18)	4.81 (4.49–5.43)	2.47 (2.30–2.72)
2033	67.75 (63.47–74.72)	4.84 (4.51–5.49)	2.48 (2.30–2.74)
2034	68.09 (63.61–75.25)	4.88 (4.53–5.54)	2.49 (2.30–2.75)
2035	68.42 (63.21–76.22)	4.92 (4.40–5.60)	2.50 (2.34–2.75)
2036	68.76 (62.93–77.18)	4.96 (4.37–5.71)	2.51 (2.32–2.79)
2037	69.10 (62.79-77.99)	5.00 (4.37–5.79)	2.52 (2.31–2.82)
2038	69.43 (62.72–78.70)	5.03 (4.37–5.87)	2.53 (2.31–2.84)

DALY, disability-adjusted life year; CI, confidence interval.
